# Integrated transcriptomic analysis and machine learning for characterizing diagnostic biomarkers and immune cell infiltration in fetal growth restriction

**DOI:** 10.3389/fimmu.2024.1381795

**Published:** 2024-09-04

**Authors:** Xing Wei, Zesi Liu, Luyao Cai, Dayuan Shi, Qianqian Sun, Luye Zhang, Fenhe Zhou, Luming Sun

**Affiliations:** ^1^ Department of Fetal Medicine & Prenatal Diagnosis Center, Shanghai Key Laboratory of Maternal Fetal Medicine, Shanghai Institute of Maternal-Fetal Medicine and Gynecologic Oncology, Shanghai First Maternity and Infant Hospital, School of Medicine, Tongji University, Shanghai, China; ^2^ Department of Gynecology and Obstetrics, The First Affiliated Hospital of Dalian Medical University, Dalian, Liaoning, China

**Keywords:** fetal growth restriction, machine-learning, immune cell infiltration, placenta, TREM1 (triggering receptor expressed on myeloid cells)

## Abstract

**Background:**

Fetal growth restriction (FGR) occurs in 10% of pregnancies worldwide. Placenta dysfunction, as one of the most common causes of FGR, is associated with various poor perinatal outcomes. The main objectives of this study were to screen potential diagnostic biomarkers for FGR and to evaluate the function of immune cell infiltration in the process of FGR.

**Methods:**

Firstly, differential expression genes (DEGs) were identified in two Gene Expression Omnibus (GEO) datasets, and gene set enrichment analysis was performed. Diagnosis-related key genes were identified by using three machine learning algorithms (least absolute shrinkage and selection operator, random forest, and support vector machine model), and the nomogram was then developed. The receiver operating characteristic curve, calibration curve, and decision curve analysis curve were used to verify the validity of the diagnostic model. Using cell-type identification by estimating relative subsets of RNA transcripts (CIBERSORT), the characteristics of immune cell infiltration in placental tissue of FGR were evaluated and the candidate key immune cells of FGR were screened. In addition, this study also validated the diagnostic efficacy of TREM1 in the real world and explored associations between TREM1 and various clinical features.

**Results:**

By overlapping the genes selected by three machine learning algorithms, four key genes were identified from 290 DEGs, and the diagnostic model based on the key genes showed good predictive performance (AUC = 0.971). The analysis of immune cell infiltration indicated that a variety of immune cells may be involved in the development of FGR, and nine candidate key immune cells of FGR were screened. Results from real-world data further validated TREM1 as an effective diagnostic biomarker (AUC = 0.894) and TREM1 expression was associated with increased uterine artery PI (UtA-PI) (p-value = 0.029).

**Conclusion:**

Four candidate hub genes (SCD, SPINK1, TREM1, and HIST1H2BB) were identified, and the nomogram was constructed for FGR diagnosis. TREM1 was not only associated with a variety of key immune cells but also correlated with increased UtA-PI. The results of this study could provide some new clues for future research on the prediction and treatment of FGR.

## 1 Introduction

Fetal growth restriction (FGR) affects 10% of all pregnancies worldwide and is a major cause of poor perinatal outcomes (1, 2). It is a condition in which a fetus does not attain its genetically conferred growth potential because of underlying pathologies, including maternal, fetal, infection, and placental abnormalities. FGR is commonly defined as the estimated fetal weight (EFW) or abdominal circumference (AC) less than the tenth percentile for the gestational age (3, 4).

The placenta is an important organ during pregnancy as the interface between fetal development and maternal circulation (5). Placenta dysfunction is a common cause of FGR, and accounts for 25%~30% of all FGR cases, which can increase the risk of iatrogenic preterm birth, very low birth weight, and poor long-term neurological prognosis (6, 7). Many major clinical problems in human pregnancy, such as FGR and preeclampsia (PE), although classically presenting in the third trimester, have their origins in the first trimester when immune regulation at the mother-fetus interface is abnormal, namely placenta dysfunction (8). Several studies have shown that immune regulation at the mother-fetus interface in embryo implantation, decidualization, and placentation (9, 10) plays an important role in the process, and is an important factor affecting the outcome of pregnancy. Furthermore, with the development of high-throughput sequencing technology, it is possible to explore the relationship between the expression of pathogenic genes and FGR caused by placental dysfunction at the transcriptome and epigenetics levels. *In vitro* and *in vivo* studies, differential expression of multiple genes have been detected through cell models and placental tissue, which may contribute to abnormal placental function through various ways, including immune regulation, and are strongly associated with pregnancy-related diseases (11–13). Additionally, given its high incidence and associated mortality rate, timely diagnosis and prediction of FGR are linked to improved outcomes (4). Various invasive diagnostic methods, such as chorionic villus sampling, have been proven effective in diagnosing and analyzing aberrant gene regulatory networks in fetuses suspected of having FGR (2). These results add new perspectives to the study of the mechanisms of FGR on one hand and provide possible clues for molecular or drug therapies targeting candidate genes and diagnostic genes to prevent and predict placenta-related diseases on the other.

Despite the exciting results of genes research in the human placenta, there is still a lack of effective diagnostic biomarkers for FGR, and our current knowledge of how numerous genes contribute to human placental development and function in pregnancy processes is still very limited. The critical role of immune factors in embryo implantation and placenta formation has also received extensive attention (14).

In this study, we used three machine learning algorithms to explore the potential biomarkers and underlying pathways involved in the development of FGR and conducted immune-related analysis of FGR placental tissue. In addition, real-world data were collected to validate the diagnostic efficacy of the biomarkers of interest identified in this study and to explore its relationship with multiple clinical parameters.

## 2 Methods

### 2.1 Data processing and identification of differentially expressed genes

GSE147776 and GSE203507 are data sets from GEO database for gene expression microarray analysis of placental tissues. GSE147776, based on the GPL20844 platform, contains 13 samples of FGR and 8 samples of normal pregnancy. GSE203507, based on the GPL16791 platform, contains 21 samples of FGR and 10 samples of normal pregnancy (**Supplementary Table 1**). By applying R packages “limma” and “sva”, we standardized the data in datasets, performed batch effect correction, and screened differentially expressed genes (DEGs). The cut-off criteria were adjusted *p* < 0.05 and | log fold change (FC)| > 1. The heatmap and volcano diagram were obtained using the “ggplots” package.

### 2.2 Enrichment analysis

Gene Ontology (GO) and Kyoto Encyclopedia of Genes and Genomes (KEGG) pathway enrichment analyses were performed by using the “clusterProfiler” and “pathview” R packages to predict the potential function of DEGs between two groups. The potential underlying molecular mechanisms of DEGs were further investigated by applying gene set enrichment analysis (GSEA). In addition, we performed disease ontology (DO) enrichment analysis of DEGs using the “Dose” R package (15).

### 2.3 Hub gene identification and construction and evaluation of diagnostic model

In this experiment, hub genes were defined as overlapping genes from three machine learning algorithms, including: least absolute shrinkage and selection operator (LASSO) regression curve with “glmnet” package (16), random forest (RF) with the “randomForest” package (17) and support vector machine model (SVM-RFE) with the “e1071” package (18). Based on the selected hub genes, we constructed a nomogram model through the “rms” package to predict the incidence of FGR. The calibration curve and receiver operating characteristic (ROC) curve were drawn to evaluate the suitability of our nomogram for clinical use (19, 20).

### 2.4 Gene set enrichment analysis and immunological correlation analysis

The “clusterProfiler” package was also used to conduct GSEA enrichment analysis to explore potential molecular mechanisms for TRMDGs. The “CIBERSORT” package was used to analyze differences in 22 immune cell infiltration levels between FGR and control groups. Box plots can show the differences in immune cells between the different groups. LASSO regression analysis was performed to identify the FGR group of candidate key immune cells from 22 types of immune cells. In addition, the correlation relationship between TREM1 and the immune cell infiltration level was analyzed and a lollipop plot was drawn to summarize the correlation between each immune cell and TREM1.

### 2.5 Validation based on the real-world data

To validate the expression changes and the prognostic value of TREM1, placenta tissues were collected from 62 singleton pregnancies with FGR and 24 singleton controls undergoing routine prenatal monitoring at the Department of Fetal Medicine & Prenatal Diagnosis Center of Shanghai First Maternity and Infant Hospital in China from January 2021 to December 2023. The clinical characteristics of patients are presented in **Table 1** and **Supplementary Table 2**. FGR was defined as birthweight below the 10th percentile for gestational age. Cases complicated with other fetal major structural and chromosomal anomalies were excluded. All placental tissues were collected during cesarean sections or spontaneous vaginal delivery. Tissue samples of approximately 1 cm^3^ from various regions near the cord attachment on the maternal side were dissected. These tissues were rinsed in saline solution to eliminate maternal contamination. A portion of the tissues was fixed in a 4% paraformaldehyde solution, embedded in paraffin, and subjected to immunohistochemistry staining (21). The remaining samples were removed the maternal side and fetal side of the placenta, snap-frozen in liquid nitrogen and stored at −80°C for subsequent RT-PCR analysis (22) and Western blot (23). Then, we also underwent immunohistochemical (IHC) staining. Detailed methods are found in the **Supplementary Materials**.

**Table 1:** The clinical characteristics of the FGR and control groups.

	Control	FGR	p-value
	(N=24)	(N=62)	
Age			
Mean (SD)	32.0 (4.2)	31.5 (4.3)	0.603
Median [Min, Max]	31.0 [24.0, 41.0]	31.0 [23.0, 42.0]	
Gestational days			
Mean (SD)	214.0 (45.9)	221.0 (37.8)	0.475
Median [Min, Max]	231.0 [139.0, 270.0]	228.0 [147.0, 269.0]	
BMI			
Mean (SD)	21.5 (2.8)	22.0 (2.9)	0.697
Median [Min, Max]	21.2 [15.2, 35.0]	21.4 [17.3, 29.0]	
Offspring gender			
Female	14 (58.3%)	25 (40.3%)	0.210
Male	10 (41.7%)	37 (59.7%)	
EFW (percentile)			
Mean (SD)	66.8 (26.4)	1.09 (2.9)	<0.001
Median [Min, Max]	75.6 [10.4, 98.9]	0.200 [0, 9.5]	
Umbilical Artery Doppler			
Normal	-	31 (50.0%)	-
Elevated PI value	-	4 (6.5%)	
AEDV	-	16 (25.8%)	
REDV	-	11 (17.7%)	
Uterine Artery PI			
Normal	–	16 (25.8%)	-
Elevated	–	46 (74.2%)	
Uterine Artery Notch			
Normal	-	31 (50.0%)	-
Notch	-	31 (50.0%)	
Pre-eclampsia			
No	–	43 (69.4%)	–
Yes	–	19 (30.6%)	

FGR, fetal growth restriction. SD, standard difference. BMI, body mass index. EFW, estimated fetal weight. PI, pulsatility index. AEDV, absent end-diastolic volume. REDV, reverse end-diastolic volume.

Partial clinical information from patients in the FGR group and the control group also were collected and the following variables for this study were extracted: age, gender of offspring, fetal weight, features of umbilical artery (UA) and uterine artery (UtA) Doppler examination in pregnancy, and the history of PE. The pulsatility index (PI) was estimated by the ultrasonography software.

TREM1 differentially expressed analysis between groups was performed using the “limma” R package. Spearman’s correlation analysis was used to describe the correlation between TREM1 expression and clinical traits. Furthermore, a ROC curve was used to assess the predictive value of TREM1 for FGR in the real world. This study was approved by the Ethics Committee of Shanghai First Maternity and Infant Hospital (Ethical number KS2133), and written informed consent was obtained from all participants before the collection of clinical samples and data.

### 2.6 Statistics analysis

**Figure 1:**
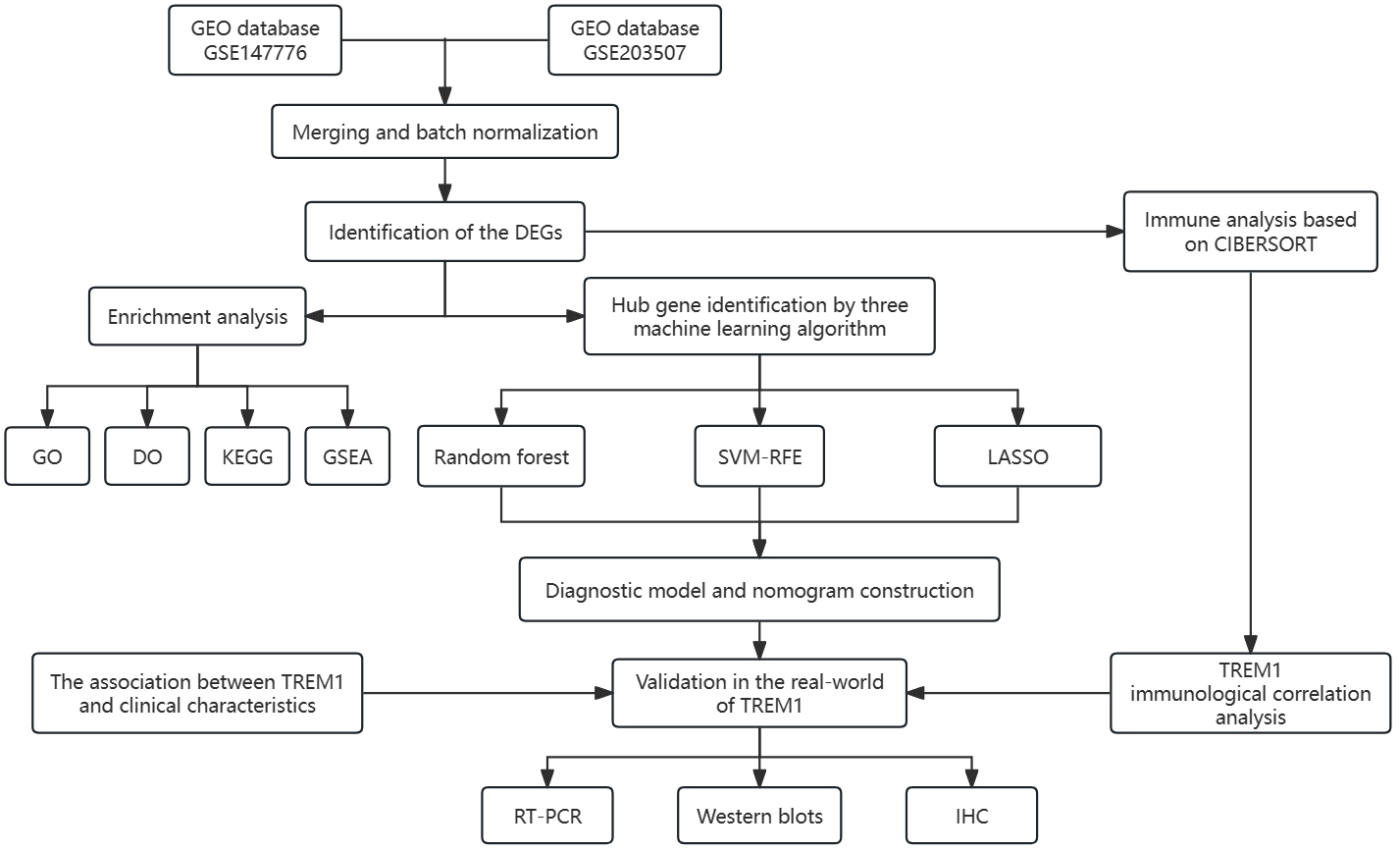
The flow chart for the whole design. DEGs, differentially expressed genes. CIBERSORT, cell-type identification by estimating relative subsets of RNA transcripts. GO, Gene Ontology; DO, disease ontology; KEGG, Kyoto Encyclopedia of Genes and Genomes; GSEA, gene set enrichment analysis; SVM-RFE, support vector machine-recursive feature elimination; LASSO, least absolute shrinkage and selection operator; IHC, immunohistochemical.

All statistical analyses were conducted using R packages [R software (version 4.2.0)]. A p-value < 0.05 was considered statistically significant (p-value < 0.001 = ∗∗∗, p-value < 0.01 = ∗∗, and p-value < 0.05 = ∗). The process and study design are presented in a flowchart (**Figure 1**).

## 3 Results

### 3.1 Identification of differentially expressed genes

**Figure 2:**
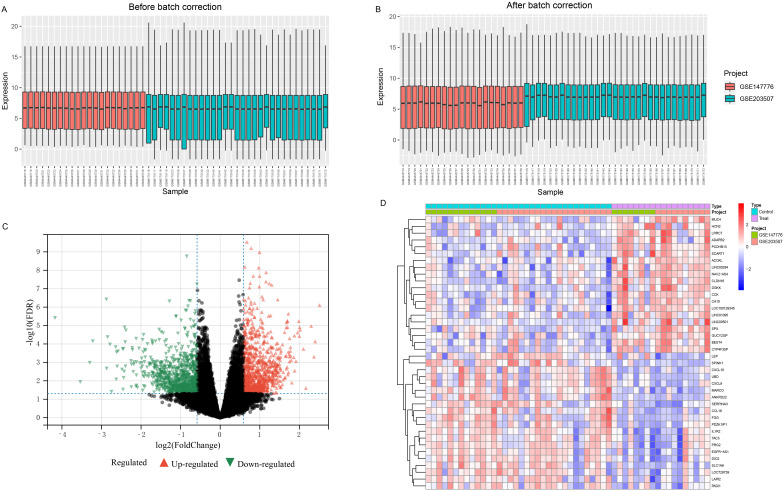
Combining different datasets and identification of DEGs. **(A, B)** Boxplots of mRNA expression distribution before and after removing batch effects. **(C, D)** DEG heatmap and volcano plot between FGR and control group. DEGs, differentially expressed genes; FGR, fetal growth restriction.

After standardization and batch effect removal of two GEO datasets (**Figures 2A, B**), a total of 290 DEGs were screened (**Figure 2C**). Top 20 up and down-regulated genes were shown in **Figure 2D**. Among them, 133 genes were up-regulated, and 157 genes were down-regulated in the FGR group (**Supplementary Table 3**). The protein-protein interaction network of DEGs was analysised through the public database STRING and we visualized the result using Cytoscape, a network visualization software (**Supplementary Figure 1**).

### 3.2 Enrichment analysis

Enrichment analysis of 290 DEGs was performed. Specifically, in the DO enrichment analysis, we found that DEGs may be related to the occurrence and development of female reproductive system diseases, asthma, pre−eclampsia and other diseases (**Supplementary Figure 2A**). The specific p-values of the DO enrichment analysis were shown in **Supplementary Table 4**. On the other hand, biological process (BP) analyses showed that leukocyte migration, regulation of cell-cell adhesion, and lymphocyte mediated immunity are the biological activities in which the DEGs are primarily involved. The result of cellular component (CC) analysis showed that the DEGs are involved in the composition of lumenal side of endoplasmic reticulum membrane, basal plasma membrane, and basolateral plasma membrane. Immune receptor activity, glycosaminoglycan binding, and cytokine receptor binding were mainly enriched according to the molecular function (MF) analysis (**Supplementary Figure 2B**). KEGG pathways analysis also indicated that DEGs are enriched in several cytokine interactions and immune-related pathways (**Supplementary Figure 2C**). GSEA analysis was performed, and the enriched pathways in the FGR group are presented in **Supplementary Figure 1D**.

### 3.3 Screening for key genes based on machine learning models

**Figure 3:**
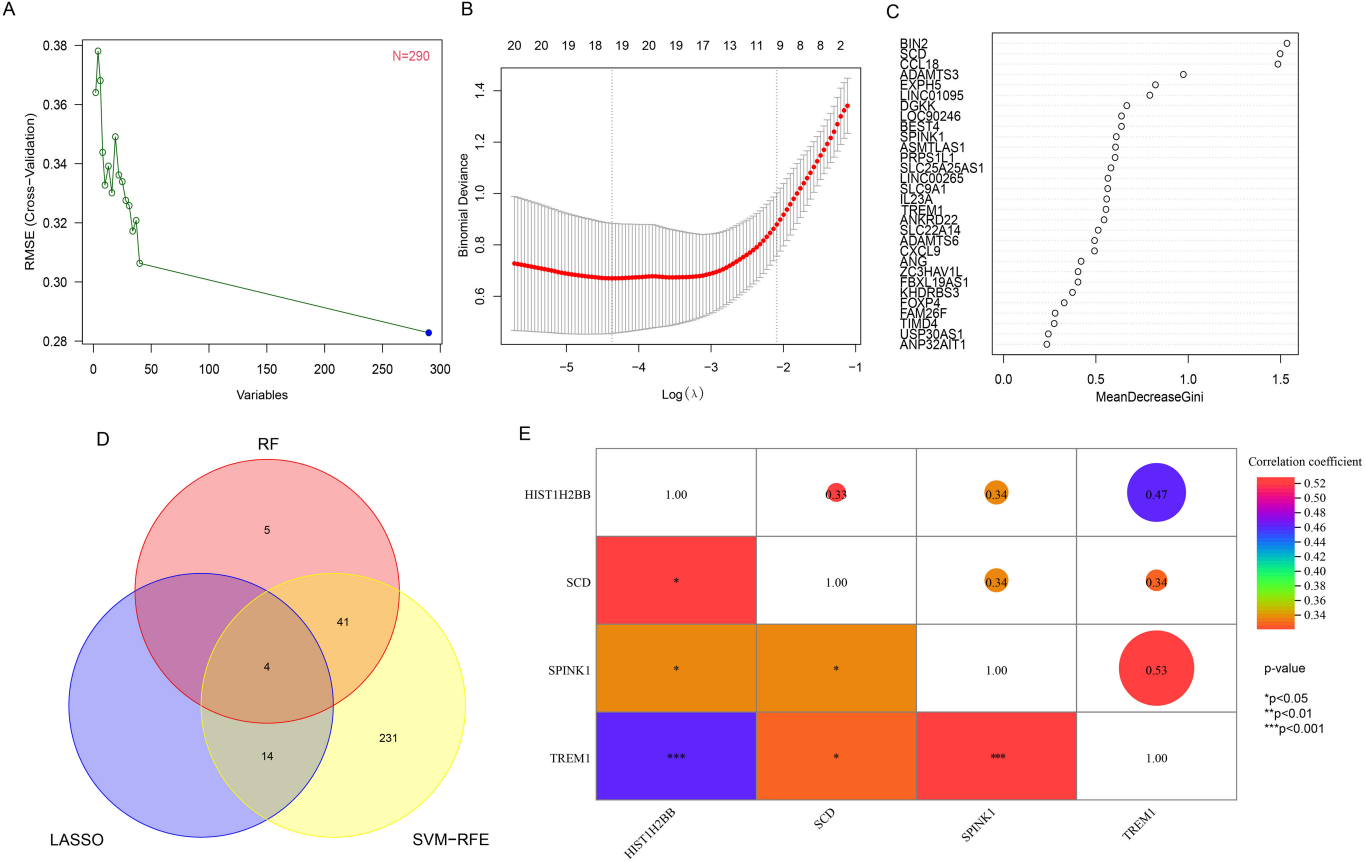
Hub gene identification and correlation analysis. **(A)** based on SVM-RFE to screen biomarkers. **(B)** LASSO logistic regression algorithm to screen diagnostic markers. **(C)** top-10 genes according to their discriminant ability in the RF algorithm. **(D)** The Venn diagram showed the intersection of diagnostic markers obtained by the results of three algorithms. **(E)** Correlation between 4 diagnostic genes. ^*^P< 0.05, ^**^P< 0.01, ^***^P< 0.001. SVM-RFE, support vector machine-recursive feature elimination; LASSO, least absolute shrinkage and selection operator; RF, random forest.

The 290 pivotal gene expression profiles were used to construct prediction functions using three machine learning models: Firstly, a total of 290 genes were screened from the DEGs via 10-fold cross-validation by SVM-RFE algorithm as diagnostic markers (**Figure 3A**). LASSO regression selected 18 predicted genes from among the statistically significant univariate variables out of candidate variables (**Figure 3B**). And 50 genes were screened from DEGs using RF and top 30 genes relative relevance was ranked from high to low (**Figure 3C**). Finally, the three algorithms identified four key genes (SCD, SPINK1, TREM1 and HIST1H2BB) with overlap (**Figure 3D**). In addition, we also found a correlation between the expression levels of four hub genes in the FGR group, and the specific correlation coefficients and p-values are displayed in **Figure 3E**. We observed significant intercorrelations among the expression levels of these four hub genes in the FGR placenta.

### 3.4 Construction of diagnostic model and developing a nomogram

**Figure 4:**
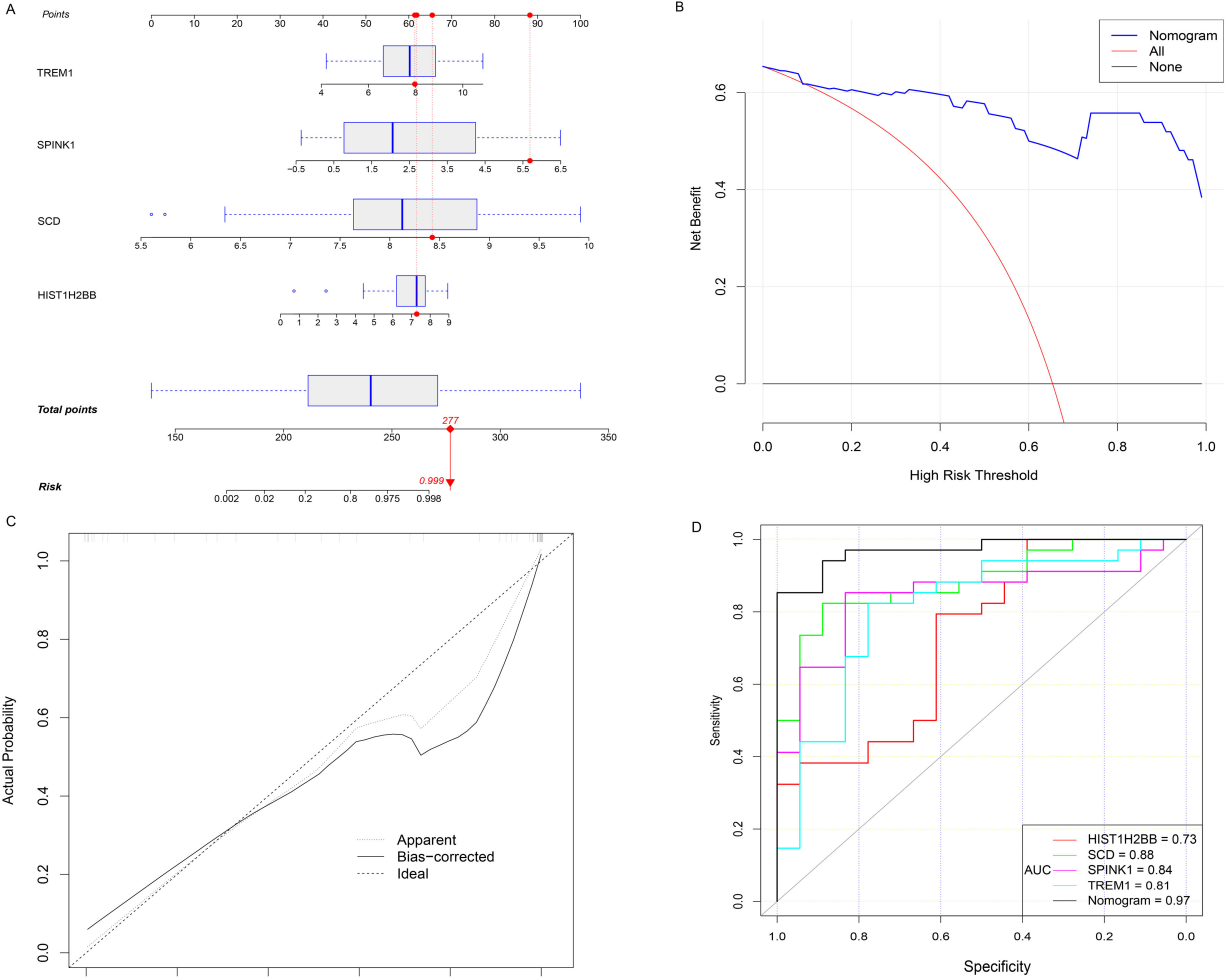
Hub genes for FGR diagnosis. **(A)** Nomogram is constructed to predict the occurrence of FGR. **(B)** DCA curves. **(C)** the calibration curves. **(D)** the diagnostic efficacy verification based on the ROC curve of nomogram and 4 hub genes respectively. OR, odds ratio; CI, confidence interval; FGR, fetal growth restriction; DCA decision curve analysis; ROC, receiver operating characteristic curve; AUC, area under the curve.

The expression levels of four key genes in the FGR group and the control group were extracted, and a multivariate Logistic regression model was constructed. The results of Logistic regression model suggested that four key genes have predictive efficacy for the occurrence of FGR (SCD, p-value < 0.001; SPINK1, p-value = 0.001; TREM1, p-value = 0.001; HIST1H2BB, p-value = 0.008) (**Supplementary Table 5**). To graphically evaluate each individual (SCD, SPINK1, TREM1 and HIST1H2BB), a nomogram was developed based on 4 key genes to predict the likelihood of FGR in each fetus (**Figure 4A**). According to the results of the decision curve analysis (DCA), the nomogram model offered a better clinical benefit. Furthermore, the calibration plot indicated that the nomogram operated in line with the ideal model (**Figures 4B, C**). The result of ROC curve showed that the prediction model based on 4 key genes had high accuracy in diagnosing FGR and the area under the curve (AUC) is 0.971 (**Figure 4D**).

### 3.5 GSEA analysis and immune characteristics analysis

GSEA results revealed the potential mechanisms of 4 hub genes and the details were presented in **Supplementary Figures 2A–D**. The results of immune cell infiltration analysis showed that the infiltration level of 22 immune cells was significantly different between the FGR group and the control group (**Supplementary Figures 5A, D**. Specifically, the infiltration level of T cells follicular helper (p-value = 0.016), NK cells activated (p-value < 0.001) and Macrophages M1 (p-value = 0.001) increased significantly in the FGR group. The level of infiltration of Macrophages M2 (p-value = 0.006) and Dendritic cells activated (p-value = 0.001) in placental tissues of FGR was significantly lower than that of control group (**Supplementary Figures 4A–E**). In order to further screen out key immune cells that may play an important role, we performed LASSO regression analysis (**Supplementary Figure 5B**) on 22 immune cells. B cells naive, B cells memory, T cells gamma delta, NK cells resting, NK cells activated, Macrophages M0, Macrophages M1, Macrophages M2 and Dendritic cells activated were selected as candidate key immune cells in the FGR group (**Supplementary Figure 5C**). The correlation between the level of infiltration of 22 types of immune cells in FGR placental tissue were also investigated and the results were shown in **Supplementary Figure 5E**.

### 3.6 Immunologic correlation analysis of TREM1

**Figure 5:**
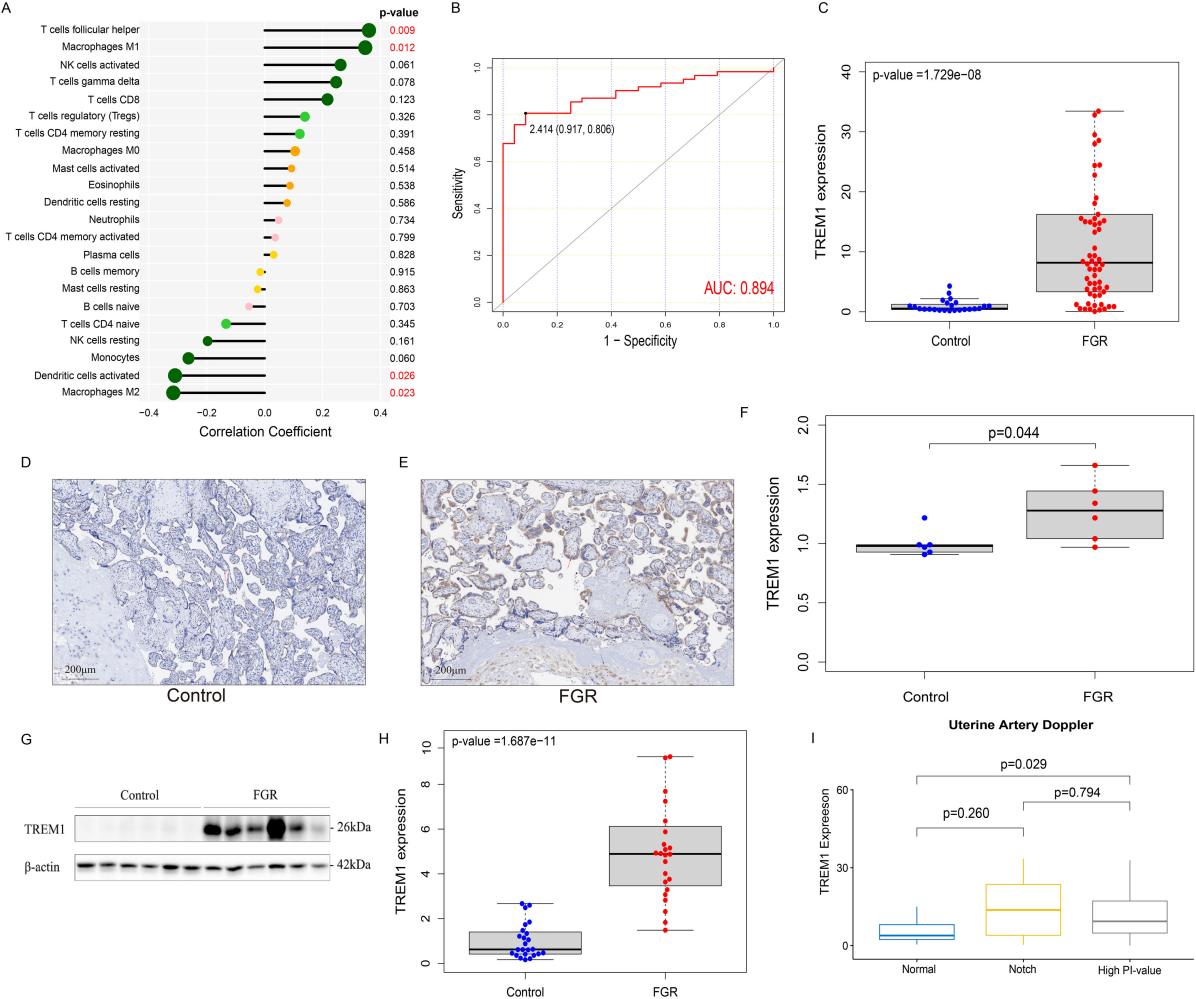
Immunologic and clinical correlation analysis of TREM1 and validation based on real-world data. **(A)** the correlation between TREM1 and immune cell infiltration in the FGR group. **(B)** the ROC curve of the diagnostic efficacy verification of TREM1. **(C)** The placental mRNA expression of TREM1 in placental tissue of normal (n=24) and FGR (n=62) fetus by real-time quantitative PCR. **(D)** IHC in the control group. **(E)** IHC in the FGR group. **(F)** The localization and expression of TREM1 in controls (n=6) and FGR (n=6) by IHC. The blue arrow points to extravillous trophoblast and the red arrow points to syntrophoblast. The blue spots represent the nuclei and brown-particle represents positive expression of TREM1. **(G)** Representative Western blot of TREM1. **(H)** The protein level of TREM1 in controls (n=24) and FGR (n=24) by western blot. **(I)** The relationship between TREM1 expression and uterine artery Doppler in the subgroup analysis. AUC, area under the curve; FGR, fetal growth restriction; IHC, immunohistochemistry; AOD, The average optical density.

In this study, we further explored the correlation between the expression level of TREM1 and 9 key immune cells and found that TREM1 was positively correlated with T cells follicular helper (Correlation coefficients: 0.36, p-value = 0.008) and Macrophages M1 (Correlation coefficients: 0.35, p-value = 0.012); and negatively correlated with Dendritic cells activated (Correlation coefficients: -0.31, p-value = 0.026) and Macrophages M2 (Correlation coefficients: -0.32, p-value = 0.023) (**Figure 5A**).

### 3.7 Validation of TREM1 in the real-world

Samples from two groups were matched based on maternal age, gestational age at delivery, gravidity and parity and the FGR singleton placentas exhibited a statistically significant increase in mRNA expression of the TREM1 gene than the normal placentas, as quantified by RT-qPCR (p-value = 1.729e−08), which was consistent with our results above (**Figure 5C**). Western blot analysis further confirmed this result (p-value = 1.678e−11) (**Figures 5G, H**). IHC revealed localization of the TREM1 protein on syntrophoblast and extravillous trophoblast (EVT). Analysis of average optical density (AOD) demonstrated a significantly higher expression intensity of TREM-1 in the FGR group compared to the normal group (p-value = 0.044) (**Figures 5D–F**).

We further explored the differences in TREM1 expression in different subgroups, including age, gender of offspring, history of PE, and features of UA and UtA Doppler examination pregnancy (**Supplementary Figures 6A–D**). The results indicated that elevated TREM1 expression in the placenta of FGR patients with UtA spectrum abnormalities (p-value = 0.043) (**Figure 5H**). Therefore, patients in the FGR group were divided into three groups: normal UtA, UtA with bilateral notch, and elevated UtA-PI. Then the analysis of differences in TREM1 expression among the three groups were performed (**Figure 5I**). We found that the TREM1 expression in patients with elevated UtA-PI is significantly higher than that in patients with normal uterine artery spectrum (p-value = 0.029). In addition, ROC curve results indicated TREM1 likelihood as valuable biomarkers in the real world (AUC = 0.894) (**Figure 5B**).

## 4 Discussion

FGR caused by placenta dysfunction is a risk factor for multiple adverse pregnancy outcomes including fetal death (24). However, to our knowledge, there is still a lack of biomarkers that can effectively predict or treat placenta-derived FGR. Therefore, in this study, we obtained DEGs by comparing gene expression levels in placental tissues of FGR group and control group. Then, three machine learning algorithms (RF model, SVM-RFE and LASSO) were used to screen out four key genes and construct a prognostic model. Whether using supervised or unsupervised techniques, machine learning algorithms can handle complex non-linear relationships, efficiently process large-scale data, automatically identify patterns and regularities, rapidly iterate and optimize models, and provide evaluations of predictive performance and accuracy (25, 26). Nomogram is also more quantitative and intuitive, which is convenient for clinicians to use (27). TREM1 has been shown to play a key role in both innate and adapted immune responses and is considered as a potential pathogenic gene for a variety of diseases (28, 29). Therefore, we took TREM1 as the focus of follow-up studies to further explore the relationship between TREM1 expression and immune cell infiltration in the FGR group. TREM1, which was identified as the potential biomarker, is highly correlated with the degree of infiltration of four immune cells (T cells follicular helper cells, Macrophages M1, DC and Macrophages M2). In addition, we also verified its expression levels and diagnostic efficacy using real-world data and explored its relationship with various clinical features.

The RF model, SVM-RFE and LASSO were subsequently screened for four key diagnostic biomarkers (HIST1H2BB, SCD, SPINK1 and TREM1). HIST1H2BB (Histone Cluster 1H2B Family Member B) encodes histone H2B type 1-b, which is a replication-dependent histone. This gene participates in the packaging of Telomere ends and RNA polymerase I promoter opening, DNA repair, transcription regulation. DNA replication plays an important role in chromosomal stability and has been shown to be a potential biomarker for high-grade serous ovarian cancer (30). However, its implication in placental diseases remains undisclosed. SPINK1 encodes a trypsin inhibitor secreted from pancreatic acinar cells into pancreatic juice, which plays an important role in various digestive systems (31). While mRNA expressing of SPINK1 was noted to be altered in mouse placenta development, its specific function in the placenta remained unknown (32). No associations with other pregnancy complications have been reported for SPINK1. SCD (Stearoyl-CoA desaturase) encodes a critical enzyme in fatty acid metabolism, catalyzing the rate-limiting step in the formation of monounsaturated fatty acids (33, 34). A previous study revealed that the inhibition of SCD attenuated the impact of oleic acid, resulting in the downregulation of migration and proliferation in human extravillous trophoblast (EVT) cells (35). Elevated placental mRNA and protein expressions of SCD were observed in gestational diabetes mellitus (GDM) pregnancies due to promoting the synthesis of palmitic acid (PA) into palmitoleic acid (POA) with anti-inflammatory effect, suggesting a potential association between SCD and the promotion of fetal growth (36, 37). Placental inflammation is often one of the main causes of FGR (38), so we hypothesized that the up-regulation of SCD expression in the placenta of FGR may be a compensatory effect against the inflammatory response. TREM1 encodes an immunoglobulin (Ig) superfamily transmembrane protein plays a key role in regulating innated and adaptive immunity. Specifically, TREM1 can multimerizes and forms a complex with transmembrane adapter TYROBP/DAP12, a SYK-mediated cascade of tyrosine phosphorylation, activating multiple downstream mediators, such as BTK, MAPK1 and MAPK3 to promote the release of pro-inflammatory cytokines and chemokines (28, 39, 40). In addition, upregulation of TREM1 expression can activate signaling pathways including Toll-like receptor (TLR) and NOD-like receptor engagement, which play a role in immune regulation (41, 42). Recently, as more researchers have begun to explore the role TREM1 plays in obstetric disorders, TREM1 have been reported to be over-expressed in the third-trimester placenta and maternal serum of PE in several studies (43–45). FGR and PE share similar pathogenesis of inadequate placentation, inflammation, and maternal vascular dysfunction (46). Furthermore, previous study also mentioned that birth weight was negatively correlated with sTREM-1 in preterm infants’ peripheral blood (47). However, no studies have reported the association between FGR and TREM1 to authors’ knowledge. Thus, in our study, we verified the mRNA and protein expression of TREM1 in second and third trimester of FGR placentas compared to gestational age-matched controls for the first time by RT-PCR, Western blot and IHC. The mRNA and protein levels of TREM1 were relatively low and stable in controls from 20 to 38 gestational weeks, while were significantly higher in FGR placentas. We also explored that the elevated mRNA levels of TREM1 was unrelated to the pregnant complicated with PE, or the maternal age, fetal gender, and umbilical artery doppler. The sole association between TREM1 overexpression and the elevated UtA-PI, along with its localization within the syntrophoblast and EVT in IHC, suggests a potential significant involvement of TREM1 in the remodeling processes of uterine spiral arteries. Studies have explored TREM1 can be both expressed in TEV-1 cells, an EVT cell line and BeWo cells, mimicking the function and phenotype of villous trophoblast (VT) cells (48). EVTs plays a dominant role in the remodeling of spiral arteries by invading spiral artery wall and replacing the smooth muscle, and finally reducing blood vessel resistance and plasticity (49, 50). However, the study manifested that the overexpression of TREM1 gene promotes migration and invasion of TEV-1 cells through activation of the NF-κB pathway (43). BeWo is the most extensively used as a cell culture model to mimic *in vivo* essentialization of placental villous trophoblast (51). The study suggested that although TREM1 did not appear to play a role in BeWo cell fusion, it is required for the induction of human chorionic gonadotropin hormone (hCG), a placental-specific protein associated with syncytialization (44). Although the upregulation of TREM1 in FGR placenta and its positive regulatory effects on EVTs seem to be contradictory, the event of placentation involves an intricate coordination of multiple cells, products, formed structures and immune system (52). TREM1 serves as a unique insight into understanding of this complex coordination under physiological and pathological conditions.

The interactions between the trophoblast cells and the maternal various immune cells have an impact on the outcome of the pregnancy (53). In the third trimester placental pathology of FGR, the expression of CD68+ Macrophages was higher, while the ratio of Macrophages M2 with anti-inflammatory effect was decreased (54), which is consistent with our findings through immune characteristics analysis. According to the results of the previous studies mentioned above and combined with the results of this study, we assume that the decrease in the number of Macrophages M2 is related to the increased production of various pro-inflammatory cytokines (55, 56), which may be related to the pathogenesis of FGR. Our study also showed increased T cells follicular helper, natural killers (NK) cells activated and decreased dendritic cells (DCs) activated in FGR placenta. in pregnant women of FGR. T cells helper cells are a special type of CD4+T cells, which can play a key role in adapted immune response by promoting B cell activation and antibody production and regulating immune memory (57). And a Single-cell RNA sequencing demonstrated that Aire+ cell depletion in pregnancy, which was thought to be a sign of causing FGR during early mouse pregnancy, results in expansion of T follicular helper cells (58). Although the specific mechanism is not clear, this result suggested that T cells follicular helper cells may be one of the causes of FGR. Studies manifested higher proportion of NK cells in umbilical cord blood (59) and reduced activation of peripheral blood DCs (60) in FGR pregnancies. DC, as an antigen presenting cell (APC), plays an important role in the development of FGR. On the one hand, DC can recognize and present fetal tissue-specific antigens in the process of placenta formation, thereby inducing maternal immune tolerance to the fetus. On the other hand, in the process of fetal growth and development, DC plays an important role in maintaining the balance of maternal immune system by regulating the activation degree of various T cells to avoid adverse effects of excessive immune response on fetal growth and development (59).

Interestingly, TREM1 was significantly positively correlated with T cells follicular helper cells and Macrophages M1, and negatively correlated with DC and Macrophages M2 infiltration levels. In addition, based on LASSO regression results, four immune cells (T cells helper cells, DCs, Macrophages M1 and Macrophages M2) overlapped with characteristic immune cells in the FGR group, suggesting that TREM1 may play a role in regulating the immune response to FGR by influencing characteristic immune cells. Overall, multiple infiltrating immune cells collectively contribute to the development of FGR, with TREM1 potentially playing a crucial role in this process.

A limitation of this study is that first trimester placenta was not available to examine the localization and over-expression of TREM-1, which cannot provide more information about how TREM-1 influence the development of human placenta in early pregnancy. Furthermore, although we found that TREM-1 was over expressed in FGR with increased UtA-PI, uterine artery measurements in these clinical cases were all at an advanced stage of the disease. There is a lack of evidence regarding the relationship between UtA-PI changes and TREM1 expression in the first trimester. This will be our next research plan to explore the predictive value of TREM-1 for early screening of FGR that mediated by placental malperfusion.

This study concluded that SCD, SPINK1, TREM1 and HIST1H2BB were diagnostic indicators of FGR by using three machine learning algorithms, and a nomogram was constructed to assess the probability of each patient developing FGR. Furthermore, the results of this study suggested that a variety of immune cells may have a role in the onset and progression of FGR, and TREM1 was not only associated with four candidate key immune cells of FGR, but also correlated with increased UtA-PI, which suggested that TREM1 may be involved in remodeling processes of uterine spiral arteries through immune regulation, thus affecting the development of FGR, and provide some new clues for our future research on the prediction and treatment of FGR.

## Data Availability

The datasets presented in this study can be found in online repositories. The names of the repository/repositories and accession number(s) can be found in the article/**Supplementary Material**.

## Supplementary material

Supplementary Figure 1 Interaction network analysis differentially expressed genes.

Supplementary Figure 2 Functional analysis of DEGs. **(A)** DO analysis. **(B)** GO enrichment analysis. **(C)** KEGG pathway enrichment analysis. **(D)** GSEA analysis. DEGs, differentially expressed genes. DO, disease ontology; GO, gene ontology; KEGG, Kyoto encyclopedia of genes and genomes; GSEA, gene set enrichment analysis.

Supplementary Figure 3 The GSEA analysis. **(A)** SCD. **(B)** SPINK1. **(C)** TREM1. **(D)** HIST1H2BB.

Supplementary Figure 4 The Violin diagram of the difference in 5 immune cells infiltration levels.

Supplementary Figure 5 Evaluation and visualization of immune cell infiltration. **(A)** PCA. **(B, C)** The LASSO technique identifies candidate key immune cells in the FGR group. **(D)** Boxplot diagram of the proportion of 22 types of immune cells. **(E)** Correlation analysis of 22 types of immune cells. PCA, Principal component analysis; LASSO, least absolute shrinkage and selection operator; FGR, fetal growth restriction.

Supplementary Figure 6 Differences of TREM1 expression and various clinical factors.

Supplementary Table 1 Information on microarray datasets obtained from GEO. GEO, Gene Expression Omnibus.

Supplementary Table 2 Detailed clinical characteristics of the FGR and control groups.

Supplementary Table 3 Details of DEGs. DEGs, differentially expressed genes.

Supplementary Table 4 Details of the disease enrichment analysis (DO).

Supplementary Table 5 The OR value and 95% CI of 4 hub genes for FGR diagnosis. OR, odds ratio; CI, confidence interval.

Supplementary Table 6 Primers of PCR.
