# Supplementary figures and images for: Integrated transcriptomic analysis and machine learning for characterizing diagnostic biomarkers and immune cell infiltration in fetal growth restriction
Source: Front Immunol. 2024 Sep 4;15:1381795. doi: 10.3389/fimmu.2024.1381795 (PMC11408188; doi:10.3389/fimmu.2024.1381795)

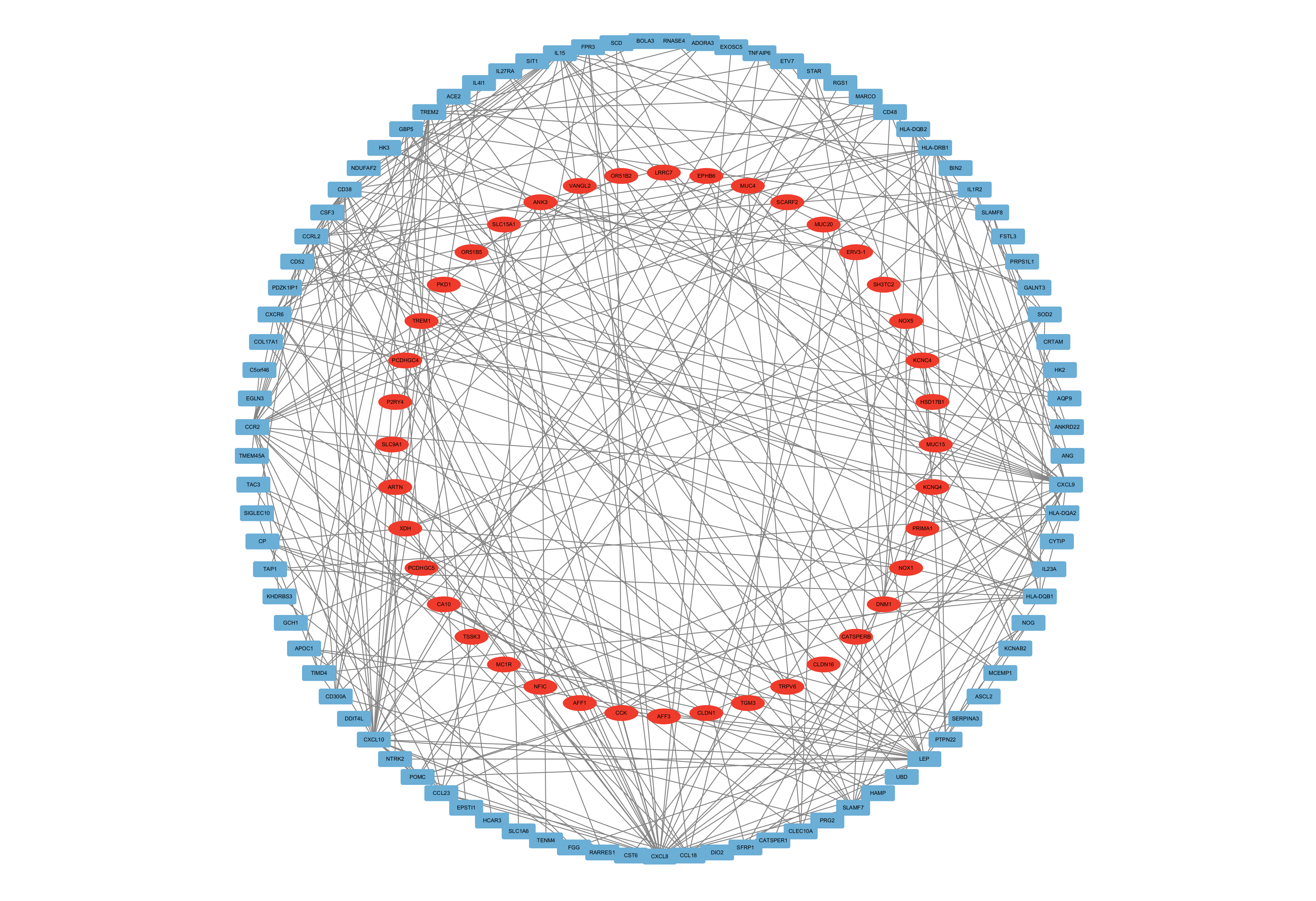

Supplement: Supplementary Figure 1 — Interaction network analysis differentially expressed genes. [file Image1.png]

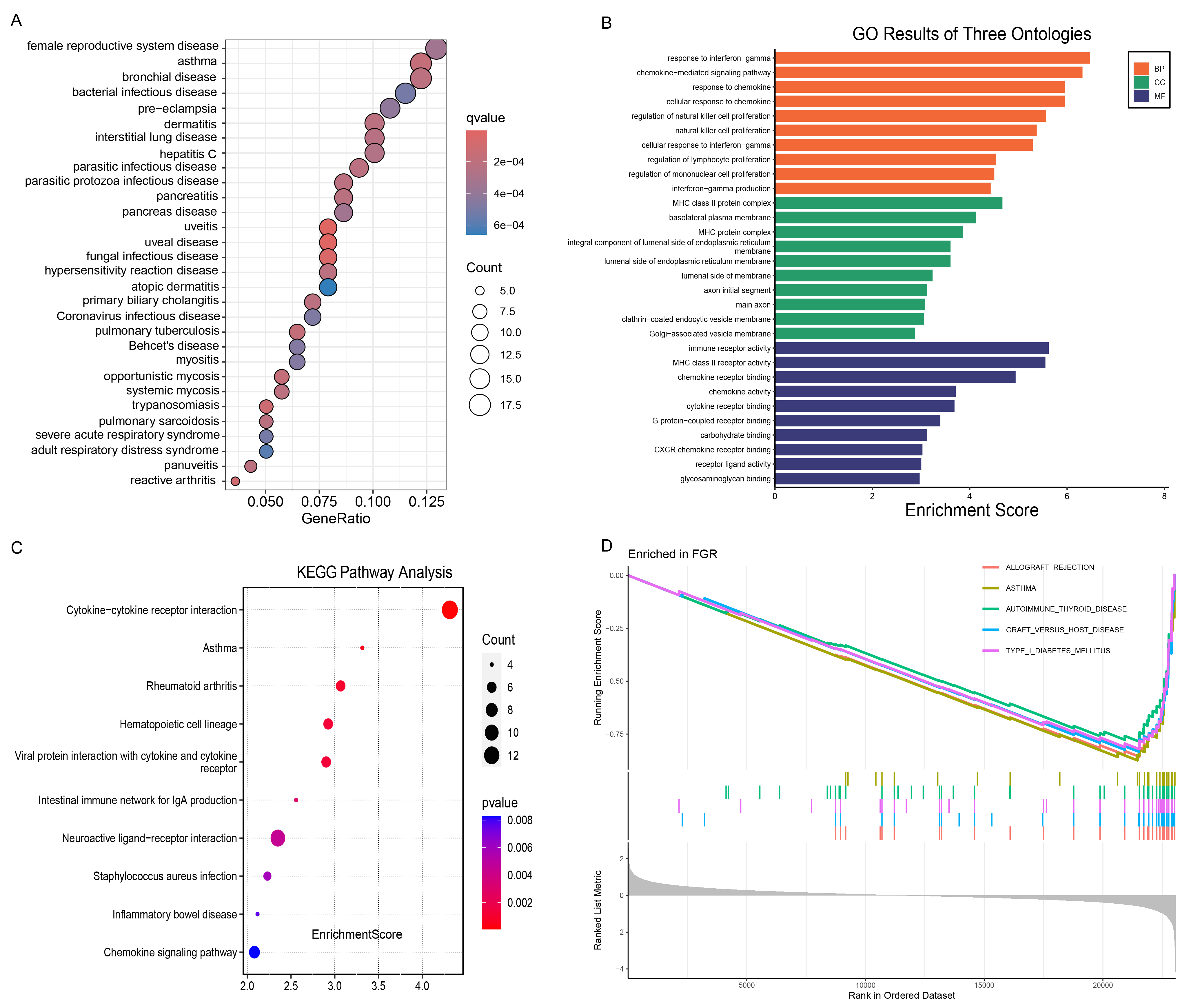

Supplement: Supplementary Figure 2 — Functional analysis of DEGs. (A) DO analysis. (B) GO enrichment analysis. (C) KEGG pathway enrichment analysis. (D) GSEA analysis. DEGs, differentially expressed genes. DO, disease ontology; GO, gene ontology; KEGG, Kyoto encyclopedia of genes and genomes; GSEA, gene set enrichment analysis. [file Image2.jpeg]

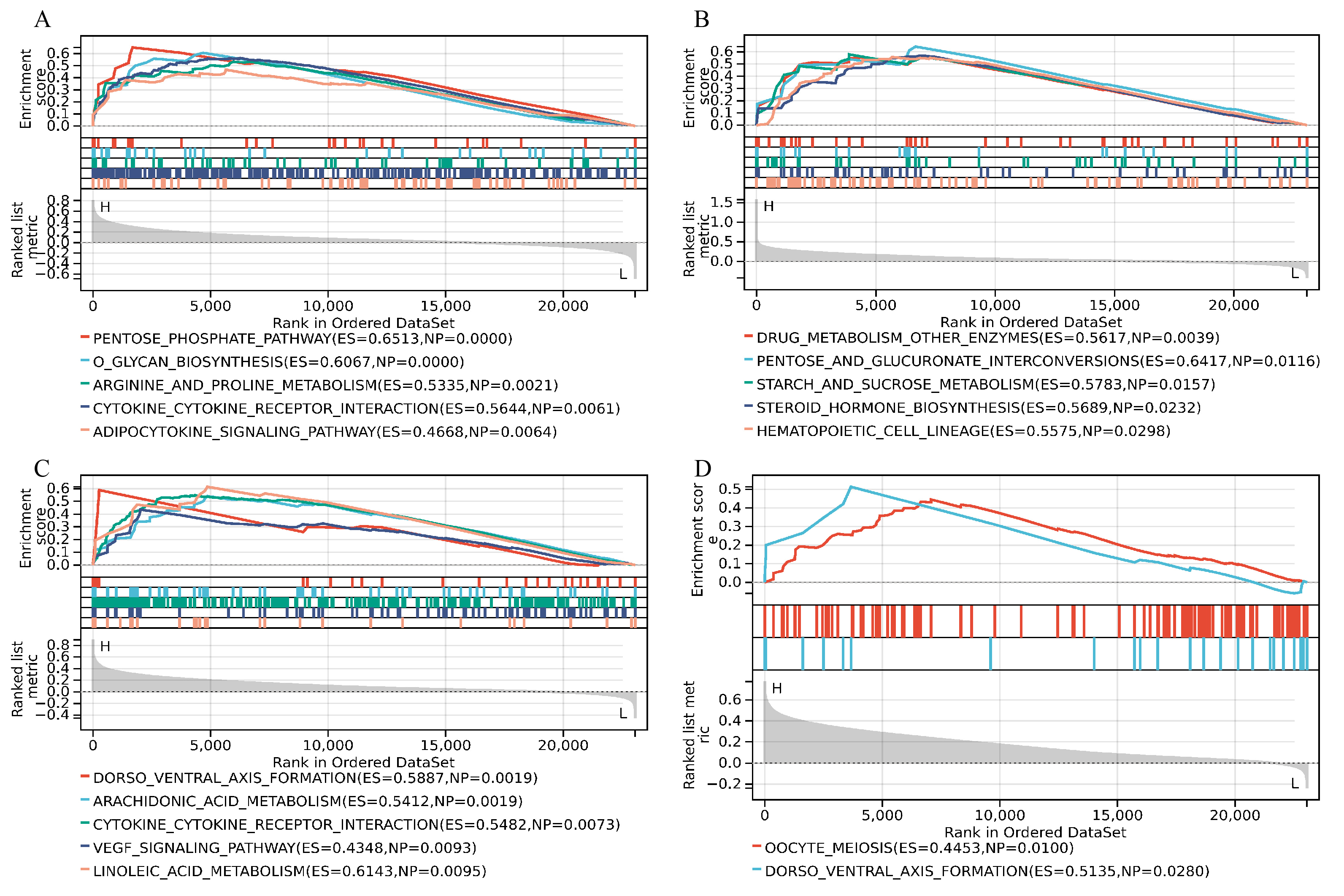

Supplement: Supplementary Figure 3 — The GSEA analysis. (A) SCD. (B) SPINK1. (C) TREM1. (D) HIST1H2BB. [file Image3.jpeg]

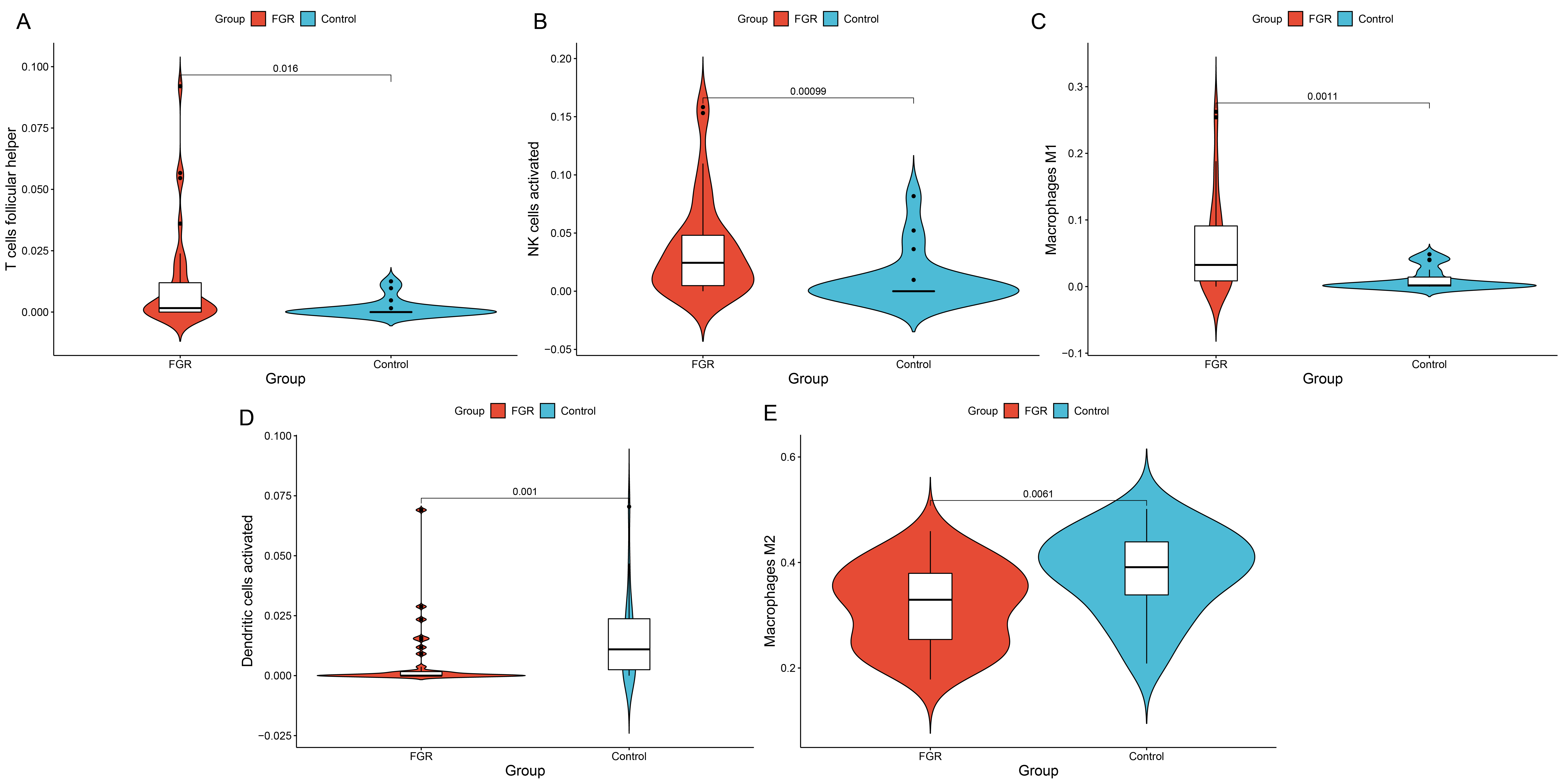

Supplement: Supplementary Figure 4 — The Violin diagram of the difference in 5 immune cells infiltration levels. [file Image4.jpeg]

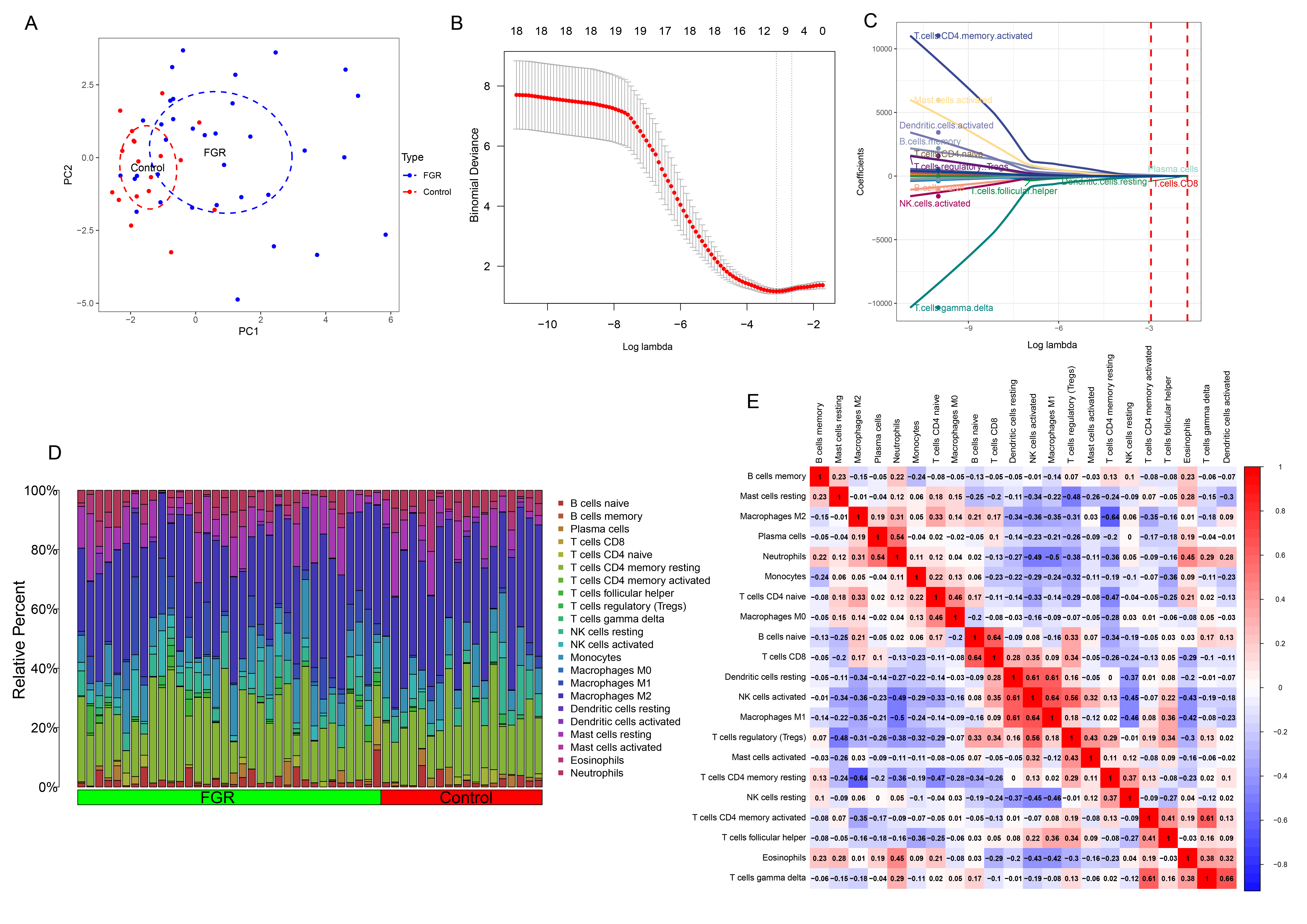

Supplement: Supplementary Figure 5 — Evaluation and visualization of immune cell infiltration. (A) PCA. (B, C) The LASSO technique identifies candidate key immune cells in the FGR group. (D) Boxplot diagram of the proportion of 22 types of immune cells. (E) Correlation analysis of 22 types of immune cells. PCA, Principal component analysis; LASSO, least absolute shrinkage and selection operator; FGR, fetal growth restriction. [file Image5.jpeg]

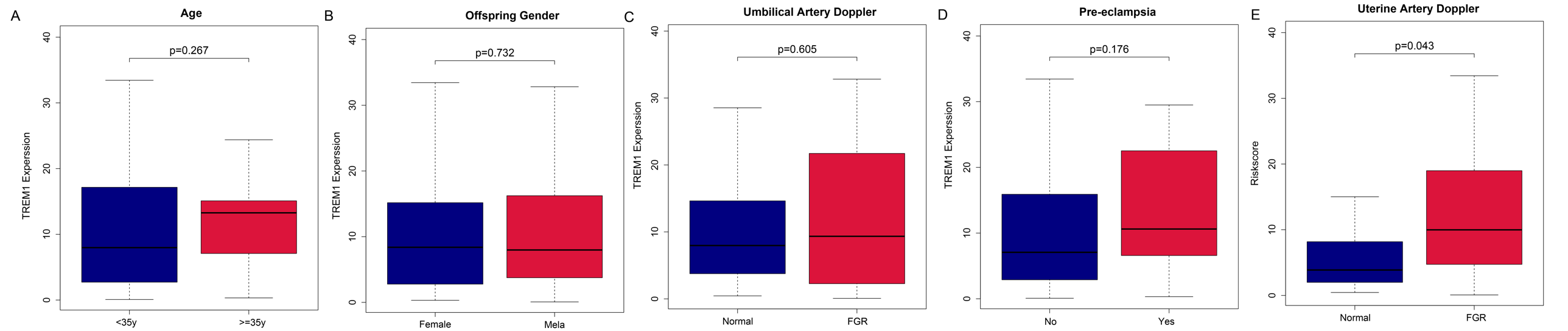

Supplement: Supplementary Figure 6 — Differences of TREM1 expression and various clinical factors. [file Image6.jpeg]
