# Supplementary material for: Integrated transcriptomic analysis and machine learning for characterizing diagnostic biomarkers and immune cell infiltration in fetal growth restriction
Source: Front Immunol. 2024 Sep 4;15:1381795. doi: 10.3389/fimmu.2024.1381795 (PMC11408188; doi:10.3389/fimmu.2024.1381795)
Supplement: Supplementary file 13 [file DataSheet1.docx]

***Supplemental Materials***

**Method**

**Real-time quantitative PCR**

Real-time quantitative polymerase chain reaction (PCR) was conducted on 62 placentas from FGR singletons and 24 placentas from control singletons. Total RNA extraction was performed using TRIzol (Invitrogen). The PrimeScript™ RT reagent Kit was employed for reverse transcription of 1ug of the total RNA within a 20ul reverse transcription reaction system, followed by reverse transcription using the PrimeScript™ RT reagent at 37°C 15 min, 85°C 5 s. Real-time quantitative PCR was performed using SYBR Green Master Mix (Takara) according to the MIQE guidelines(1). The reaction was on the 384-well optical reaction plate Applied Biosystems 7500 Real-Time PCR System. As per the manufacturer's protocol, 20 μg cDNA was used in 10 ul qPCR reaction system. The reaction parameters consisted of an initial step 95°C for 30 seconds, followed by forty cycles of 5 seconds at 95°C, 30 seconds at 56°C and 30 seconds at 72°C. The quantitative real-time PCR experiment was performed in triplicate. Primer sequences used are detailed in the Supplement Table 6. The comparative Ct (2−ΔCt) method was used to determine RNA values of the TREM1 gene, with the expression of the reference gene ACTB detected in placenta samples.

**Western Blot**

24 FGR and 24 control placenta tissues were harvested in RIPA (Epizyme) lysis buffer supplemented with protease inhibitors (Epizyme). For Western blot analysis, 20 micrograms of protein per well was loaded onto SDS polyacrylamide gels and subsequently transferred onto PVDF membranes. After blocking with 5% nonfat dry milk in TBST for 1 hour, the membranes were incubated overnight at 4°C with TREM1 antibody (1:1000 dilution, R&D), followed by incubation with an anti-goat HRP-conjugated secondary antibody (1:3000 dilution, Servicebio) for 1 hour at room temperature. The internal reference protein used was β-actin (1:50000 dilution, Proteintech), and it was probed using an anti-mouse HRP-conjugated secondary antibody (1:3000 dilution, Beyotime). The blots were visualized using ImageJ software.

**Immunohistochemistry (IHC)**

Following the previously established protocol, we performed immunohistochemical staining on placental tissues from 6 FGR cases and 6 controls to assess TREM1 protein levels(2). Immunostaining utilized an immunohistochemistry kit (Dako, Denmark). Paraffin-embedded placental tissues were sectioned, dewaxed, and hydrated using xylene and ethanol. To inhibit endogenous peroxide activity, sections were treated with a blocker for 10 minutes. Heat-mediated antigen retrieval was performed with Tris-EDTA buffer (pH 9.0) for 20 min. Subsequently, sections were blocked with 5% bovine serum albumin at room temperature for 35 minutes. Tissue sections were incubated with primary antibody TREM1 (1:150 dilution, R&D) overnight at 4°C, followed by incubation with an anti-goat HRP-conjugated secondary antibody for 35 minutes at 37°C. Antibody binding was detected using diaminobenzidine (DAB) color developing solution, and sections were counterstained with the hematoxylin. After dehydration, the sections were sealed and observed. Images were captured using a microscope and a digital slice scanner (3DHISTECH, Hungary). The presence of brownish yellow granules in the cytoplasm or nucleus was considered indicative of positive staining. Five non-overlapping areas of the same size were chosen for analysis by ImageJ software. Mean values for area-integrated optical densities were assessed. The average optical density (AOD) was used to represent the expression intensities of TREM1.

**Reference**

1. Bustin SA, Benes V, Garson JA, Hellemans J, Huggett J, Kubista M, et al. The MIQE guidelines: minimum information for publication of quantitative real-time PCR experiments. Clinical chemistry. 2009;55(4):611-22.

2. Sun L, Zhou J, Wang K, Wang J, Shang L, Zhang J, et al. Placental Up-Regulation of Leptin and ARMS2 is Associated with Growth Discordance in Monochorionic Diamniotic Twin Pregnancies. Twin research and human genetics : the official journal of the International Society for Twin Studies. 2017;20(2):169-79.
